# Impact of hand osteoarthritis in women on maximal forces in six different grasp types

**DOI:** 10.1038/s41598-023-39198-z

**Published:** 2023-09-04

**Authors:** Verónica Gracia-Ibáñez, Néstor J. Jarque-Bou, Vicente Bayarri-Porcar, Alba Roda-Sales, Pablo Granell, Margarita Vergara, Joaquín L. Sancho-Bru

**Affiliations:** 1 Department of Mechanical Engineering and Construction, Universitat Jaume I, Av. Vicent Sos Baynat, s/n, 12071 Castelló, Spain; 2 Consorci Hospitalari Provincial de Castelló, Av. del Dr. Clarà, 19, 12002 Castelló, Spain

**Keywords:** Bone quality and biomechanics, Biomedical engineering

## Abstract

This work aims to: (1) Provide maximal hand force data on six different grasp types for healthy subjects; (2) detect grasp types with maximal force significantly affected by hand osteoarthritis (HOA) in women; (3) look for predictors to detect HOA from the maximal forces using discriminant analyses. Thirty-three healthy subjects (37 ± 17 years, 17 women, 16 men) and 30 HOA patients (72 ± 9 years, all women) participated in the experiment. Participants were asked to exert their maximal force while performing six different grasp types 3 times. Two MANOVAs were conducted to detect if force depended on gender in healthy participants and if force significantly diminished in women with HOA. Finally, a linear discriminant analysis for detecting HOA was performed using forces of the grasp types that were significantly affected by HOA. Gender-disaggregated statistics are provided for healthy participants. Significant differences are obtained for all grasp types per gender. The women with HOA exerted significantly lower force values (*p* < 0.001) for all the grasp types than healthy ones. The discriminant analysis revealed that oblique grasp was the most significant one for detecting HOA. A discrimination equation was obtained with a specificity of 88.2% and a sensitivity of 83.3%. This work provides grip force data on six grasp types for healthy participants and for women with HOA. HOA women present reduced strength in all grasps due to pathology. Three of these grasps are a novelty. Oblique grasp strength may suffice to discriminate a patient with HOA, which might help non-invasive HOA detection.

## Introduction

Maximal force capabilities of injured or pathological hands are often compared to those of healthy hands to assess the impact of either injury or disease on hand function. This comparison is done by measuring pinch and cylindrical grasp force capabilities. In clinics, whenever possible the force capability of the injured hand is compared to that of the non-injured one using the ‘10% rule’ because of the existing difference between the dominant and non-dominant hand^1^. When both hands are affected by injury or disease, normative values are used to make a comparison. Normative data (cylindrical and pinch maximal forces) for adults, segregated by gender and age, were firstly provided by Mathiowetz et al.^2^ according to the standardised procedures of the American Society of Hand Therapists for hand and arm positioning^3^. Cylindrical and pinch maximal grasp forces are significantly higher for males, remain quite stable from 20 to 59 years, but gradually decline from 60 to 79 years^2^. The mean declining right hand force of healthy women aged over 60 years versus those under 60 years is 29% for cylindrical grasp and 16% for pinch grasp. These maximal force capabilities do not depend on arm position^4^, but on ethnic differences based on upper limb anthropometry^5^. Normative values for different ethnicities disaggregated by gender and ages can be found in the literature^6–9^. Dynamometry is considered the gold standard for grip strength assessment due to its portability, real-time response, and wide availability in both clinical and research settings. Pressure mapping systems are also used in research, especially when the aim is to analyse the pressure distribution rather than to provide a normative value of force under normal conditions. A recent study suggests their use for recording grip forces^10^, but their cost and complexity make them unfeasible for clinical practice.

**Figure 1:**
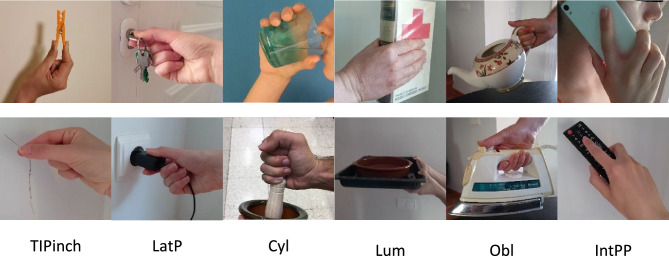
Images of the grasps applied for performing common activities of daily living. TIPinch: thumb-index pad-to-pad pinch; LatP: lateral pinch, where the lateral part of the index finger is used, and also the thumb; Cyl: cylindrical grasp, where the palm is involved, and the thumb is in direct opposition to fingers in abduction or neutral; Lum: lumbrical grasp, where the thumb and the proximal part of fingers are involved, but the palm is not; Obl: oblique palmar grasp as a variation of the cylindrical grasp and the palm is involved, but the thumb is adducted; IntPP: Intermediate power-precision grasp, where the palm is somewhat involved, but both the thumb and index finger stabilise the grasp.

The ability to grasp is key for ensuring patients’ autonomy. Performing activities of daily living (ADLs) requires grasping objects and applying forces on them to be able to do tasks. The applied grasping postures depend on the object to be grasped and on the task to be performed, and are often categorised using different taxonomies^11–13^. In this paper, the taxonomy of^13^ is applied (Fig. 1): thumb-index pad-to-pad pinch (TIPinch), lateral pinch (LatP), cylindrical grasp (Cyl), lumbrical grasp (Lum), oblique palmar grasp (Obl) and intermediate power-precision grasp (IntPP). The daily frequency of these grasp types has been analysed in a previous study^13^, as has their relevance for functionality^14^. In addition to the pad-on-pad pinch grasp, other types of grips were demonstrated to be important. While the pad-on-pad pinch grasp is the most common and significant, it is closely followed by cylindrical, lateral, lumbrical, and oblique grasps, which are used with comparable frequencies depending on the hand and whether it is used individually or simultaneously. In terms of significance, lateral and oblique grasps are slightly less relevant than cylindrical and lumbrical grasps. However, only normative data for cylindrical grip and pinch (thumb-index pad-to-pad pinch and key or lateral pinch) are available in the literature. For other grasp types, the data reported in literature are scarce and focus on specific tasks. For instance, maximal forces of 24 female workers during manual insertions in automotive manufacturing using oblique grasp are reported in^15^.

Hand osteoarthritis (HOA) is a highly prevalent chronic disease that affects 67% of women and 55% of men aged over 55 years^16^. HOA symptoms include pain, stiffness and inflammation, which narrow ranges of movement^17^ and reduce grasp force^18^ in pinch and cylindrical grasps. HOA patients aged under 64 years develop significantly smaller maximal cylindrical force^18^ compared to the values segregated by gender and age of Mathiowetz et al.^2^, with a mean decrease of over 25% for patients (male and female) aged less than 60 years, of about 13% for female patients aged 60–64 years, and no significant decrease for patients (male and female) aged over 64 years. The inclusion criteria for the participants aged > 60 years in Mathiowetz et al.^2^, age at which a decline in force is observed, were: do not experience acute pain and self-report as being able to perform a normal lifestyle. Hence the decrease reported by Mathiowetz et al.^2^ for women over the age of 60 might be due to degenerative diseases like HOA, especially when considering that almost no differences appear between women aged > 60 with and without HOA^18^ (only a minor difference for those aged between 60 and 64 years).

The problems faced by HOA patients depend on circumstances, and the impact on their daily lives vastly varies, and frequently in accordance with socio-economic conditions and psychological support^19^. HOA patients encounter lack of understanding the impact of HOA on their daily lives^20^, and they claim that some tasks are limiting, such as those requiring force and fine manipulation^17,21,22^, especially wringing out washcloths, opening jars or doing up buttons^22^, using a toothbrush, cutting food with a knife^21^ or removing heavy trays from ovens. Functional tests like the Sollerman Hand Function test are of no avail to reflect these problems^17^. We hypothesise that the assessment of the grasp force capabilities (maximal grasp forces) of HOA patients for different relevant grasp types might help to better understand the hand function difficulties encountered by patients, and to also better decide specific rehabilitation processes^19^. In addition, those maximal grasp forces significantly affected by HOA might be considered to be potential predictors for detecting the disease, analogously to the use of altered ranges of motion proposed in a previous study as a non-invasive detection method^23^. These tests may serve as non-invasive tools to point out the need for imaging diagnosis.

In this paper the maximum achievable forces using six different grasp types were measured in two samples, healthy and HOA subjects, and in both dominant and non-dominant hands, to: (1) provide normative data for healthy subjects, disaggregated by gender on six grasp types relevant in ADLs (lumbrical, intermediate power-precision and oblique grasps as a novelty, and cylindrical grasp, thumb-index pad-to-pad pinch and lateral pinch) being three of them a novelty; (2) detect grasp types significantly affected by HOA in maximal force loss terms to identify the daily tasks potentially hindered in HOA patients because of using hampered grasp types; (3) look for predictors to detect HOA by using the maximal forces of hindered grasp types in discriminant analyses so that they can serve clinicians as a further tool to decide convenience of image diagnosis.

## Methods

The healthy sample (sample H) consisted of 33 right-handed subjects free of hand diseases (37 ± 17 years, 17 women, 16 men). The HOA sample (sample P) comprised 30 HOA right-handed patients (72 ± 9 years, all women). All the participants signed written consent to participate in the experiment. Patients were recruited by clinicians from among hospital patients exhibiting various disease stages and distinct levels of compromise, but none had undergone surgery.

**Figure 2:**
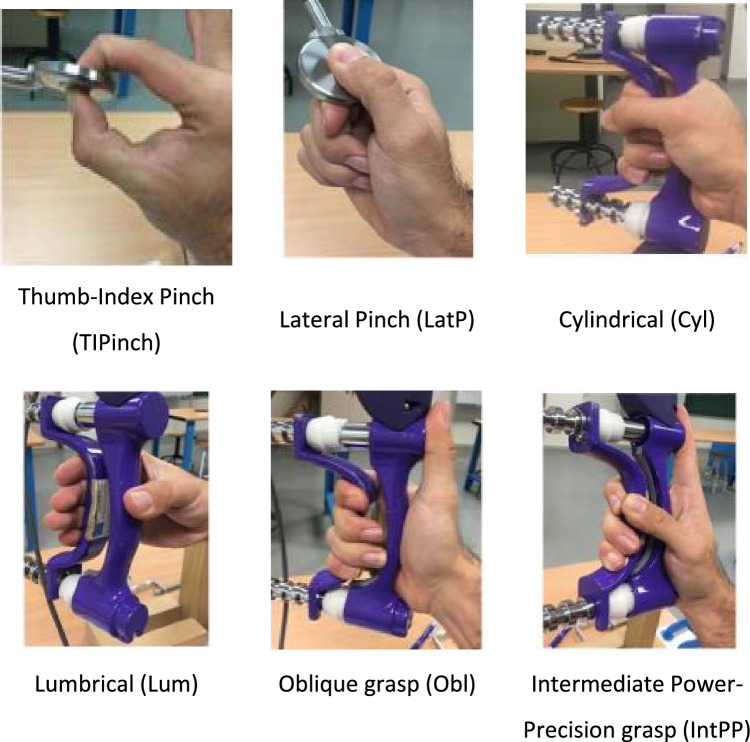
Six grasp types whose maximal force was recorded. Grasp type definitions according to^12^.

According to the standardised method established in^2^, participants were seated with their shoulder adducted and neutrally rotated, elbow flexed at 90°, forearm in a neutral position, and wrist between 0° and 30° dorsiflexion and between 0° and 15° ulnar deviation. Six different grasp types were evaluated (Fig. 2): thumb-index pinch and lateral pinch were performed with a Biometrics Pinchmeter (Biomemetrics Ltd.); cylindrical, lumbrical, oblique and intermediate power-precision grasps were performed with a Hand Grip Biometrics Dynamometer (Jamar) in the second handle position. Upon hearing the practitioner’s voice, participants exerted the maximal force and maintained it for 3 s^24^.

Prior to each grasp, images of the grasp applied in common ADLs (Fig. 1) were shown to participants so they could familiarise with the requested task. The grasp types order was randomised between participants. To ensure test–retest reliability^24^, each participant performed three repetitions of each grasp type with both hands. Starting with their right hand, resting for at least 30 s, following with their left hand, and resting again before the next repetition. Thus a minimum rest period of 1 min was assured. Each record was trimmed to 2 s when maintaining maximal force, and the mean value during the 2 s was taken as the maximal force per repetition.

Intrasession repeatability and reliability were analysed because they were only checked in previous studies for cylindrical, thumb-index pinch and lateral pinch^24^. An intrasession repeatability analysis was firstly performed for each grasp type in every sample by means of a set of ANOVAs with force as the dependent variable and the participant as the factor. The precision error was calculated as the square root of the quadratic mean within groups, representing the variability that cannot be attributed to the factor. Despite not being the most appropriate way to measure reliability, the intraclass coefficient (ICC) and correlation coefficients (Pearson’s coefficient (Pcc for normal distributions, Spearman’s otherwise) were computed to make a comparison to the reliability data provided in the literature. In addition, a repeated measures ANOVA per hand and grasp type was performed, with force as the dependent variable and repetition as the factor. The repeated measures ANOVA was used to check whether fatigue could have an effect on the maximal force exerted, i.e., lowering force in the three subsequent repetitions.

The maximal forces measured in the three repetitions were averaged. Statistics were obtained for both samples and were also segregated by gender for the healthy sample: subsample H_w (17 women, 37 ± 13 years) and subsample H_m (16 men, 37 ± 13 years). Then two MANOVAs were run with force in each grasp type as the dependent variables, and sample and hand as factors. The first MANOVA compared samples H_w and H_m to assess the effect of gender. The second MANOVA compared sample P and subsample H_w to identify which grasp types were hindered by HOA in maximal force loss terms. The decrease in the percentage of the mean force value of women with HOA with respect to healthy ones was also computed.

The percentiles of the force values per grasp and hand of the patients as if they belonged to sample H_w were computed and histograms represented to assess the decrease in force and understand the distribution of this reduction.

Finally, the force values of the grasp types that significantly differed between patients (sample P) and healthy women (subsample H_w) were used as the independent variables in a linear discriminant analysis performed in SPSS 28 to find a discriminant function to detect HOA in women with the smallest set of predictor variables. Condition (patients or healthy participants) was taken as the categorical variable. The stepwise method was followed: predictor variables entered sequentially. In particular, Wilks’ lambda was used, which checks how well each potential predictor variable contributes to the function: 0 means total discrimination, while 1 denotes no discrimination. Each potential predictor variable is tested by placing it in the function and then taking it out to generate a Λ statistic. The significance of the change in Λ is measured by an F-test. The variable is entered in the function if the significance level of its F-testis lower than the entry value (0.05) and is removed if the significance level is higher than the removal value (0.1). The goodness of the classification ability was checked by leave-one-out cross validation, which repeats the analysis by taking one case out in each repetition. Thus, in the cross validation, the detection method’s sensitivity is obtained as the percentage of women who truly have the disease and are correctly identified, whereas specificity is obtained as the percentage of those who truly do not have the disease and are correctly identified. This analysis was performed twice (one for the right hand and one for the left hand) where the maximal force of those grasp types for which there was a significant difference between patients and healthy women was taken as the initial entry variables. The discriminating ability of the function was also checked with its eigenvalue and canonical correlation.

### Ethics approval and consent to participate

This study was performed in line with the principles of the Declaration of Helsinki. Approval was granted by the *Scientific Committee*/‘*Consorcio Hospitalario Provincial de Castellón, Spain*’ approval to the non-invasive clinical experiment and by the *Research Ethics Committee with Human Beings (formerly Deontology Committee*) */’Universitat Jaume I, Spain*”**,** reference numbers CD/31/2019 and CD/27/2022. Informed consent was obtained from all individual participants included in the study.

## Results

The intrasession errors (Table 1) ranged between 7 and 15% for healthy subjects and between 12 and 21% for patients and were similar for the dominant and non-dominant hands. The ICC (Table 1) obtained for HOA patients ranged from 0.94 to 0.97 and from 0.92 to 0.98 for healthy participants. Table 2 shows the Pearson Correlation Coefficients, which ranged from 0.80 (lateral pinch, right hand) to 0.94 (cylindrical and intermediate power-precision grasps, left hand and oblique grasp, right hand) for HOA patients, and from 0.73 (lumbrical and thumb-index pinch grasps, right hand) to 0.96 (cylindrical and lateral pinch grasps, right hand) for healthy participants.

**Table 1:** Intra-session repeatability: Precision error in kg, not explained by factor (ANOVA per grasp type factor Participant) and the percentage in brackets refers to each mean value and Intraclass coefficient (ICC).

		GRASP PRECISION ERROR in kg and in % in brackets—ICC											
		Cyl		IntPP		LatP		Lum		Obl		TIPinch	
	Hand	Error	ICC	Error	ICC	Error	ICC	Error	ICC	Error	ICC	Error	ICC
Sample P	Right	1.98 (13%)	0.96	1.10 (13%)	0.97	0.59 (18%)	0.95	0.71 (15%)	0.95	1.96 (20%)	0.95	0.35 (18%)	0.96
	Left	1.51 (12%)	0.97	1.40 (18%)	0.95	0.44 (15%)	0.96	0.55 (12%)	0.96	2.01 (21%)	0.94	0.37 (19%)	0.94
Sample H	Right	3.05 (9%)	0.97	2.21 (15%)	0.92	0.49 (7%)	0.98	0.81 (10%)	0.92	3.29 (12%)	0.97	0.56 (13%)	0.92
	Left	3.08 (10%)	0.97	1.19 (9%)	0.97	0.45 (7%)	0.98	0.59 (8%)	0.97	2.76 (11%)	0.97	0.47 (12%)	0.93

*Cyl* cylindrical, *IntPP* intermediate power-precision, *LatP* lateral pinch, *Lum* lumbrical, *Obl* oblique, and *TIPinch* thumb-index pinch.

**Table 2:** Intra-session repeatability: Pearson correlation coefficients (Pcc) or Spearman correlation coefficients (Scc) depending on the normality of the distribution.

		Correlation coefficients between repetitions Ri-j (i, j = 1, 2, 3). (*)																	
		Cyl			IntPP			LatP			Lum			Obl			TIPinch		
		R1-2	R1-3	R2-3	R1-2	R1-3	R2-3	R1-2	R1-3	R2-3	R1-2	R1-3	R2-3	R1-2	R1-3	R2-3	R1-2	R1-3	R2-3
Sample P	Right	0.90	0.86	0.93	0.91	0.91	0.94	0.86	0.80	0.92	0.91	0.90	0.90	0.89	0.87	0.94	0.92	0.91	0.91
	Left	0.94	0.91	0.93	0.90	0.85	0.86	0.87	0.91	0.93	0.88	0.93	0.85	0.89	0.81	0.82	0.81	0.84	0.90
Sample H	Right	0.92	0.91	0.96	0.89	0.81	0.76	0.96	0.93	0.96	0.73	0.81	0.86	0.87	0.86	0.88	0.83	0.73	0.81
	Left	0.92	0.90	0.95	0.91	0.89	0.93	0.93	0.94	0.95	0.89	0.89	0.95	0.88	0.90	0.92	0.83	0.75	0.88

Grasp types abbreviations are explained in the caption of Table 1.

(*) underlined those distributions that are not normal (Kolmogorov–Smirnov). In these cases, the Spearman correlation coefficient is shown instead of the Pearson correlation coefficient.

Significant differences (*p* < 0.05) between repetitions in the repeated measures ANOVA were found for the HOA patients’ sample in right hands for the oblique and lumbrical grasps. However, the mean value of the third repetition was higher. For the healthy sample, significant differences were found for right hands for intermediate power-precision and lateral pinch grasps. In this case the mean value of the second repetition was higher for the intermediate power-precision grasp, and the value increased with the repetitions for lateral pinch grasp.

Table 3 shows the statistics of the maximal forces measured for each grasp type and for the two samples. The healthy participants subsample is also disaggregated by gender.

**Table 3:** Statistics of the force values per grasp. Grasp types abbreviations are explained in the caption of Table 1.

			FORCE (KG)					
	Hand	Statistic	Cyl	IntPP	LatP	Lum	Obl	TIPinch
Sample P	Right	Mean (SD)	14.96 (5.81)	8.29 (3.70)	3.37 (1.48)	4.56 (1.83)	9.99 (4.89)	1.98 (1.06)
	Left	Mean (SD)	13.00 (5.19)	7.77 (3.63)	3.07 (1.35)	4.40 (1.55)	9.46 (4.74)	1.96 (0.90)
Sample H	Right	Mean (SD)	33.83 (10.87)	14.84 (4.48)	6.73 (1.93)	8.05 (1.61)	28.44 (10.38)	4.43 (1.13)
	Left	Mean (SD)	29.82 (10.58)	12.80 (3.81)	6.07 (1.87)	7.32 (1.84)	25.78 (9.62)	4.07 (1.04)
Subsample H_m	Right	Mean (SD)	42.20 (8.84)	17.83 (4.14)	8.21 (1.29)	8.92 (1.45)	35.06 (10.76)	4.88 (0.97)
	Left	Mean (SD)	37.58 (9.19)	15.47 (3.13)	7.54 (1.31)	8.45 (1.54)	32.10 (9.75)	4.36 (0.99)
Subsample H_w	Right	Mean (SD)	25.96 (5.22)	12.02 (2.60)	5.33 (1.28)	7.24 (1.33)	22.20 (4.65)	4.00 (1.13)
	Left	Mean (SD)	22.51 (5.26)	10.28 (2.42)	4.69 (1.10)	6.26 (1.44)	19.83 (4.31)	3.80 (1.03)

As expected, the first MANOVA results obtained by comparing H_w and H_m showed that gender significantly affected healthy subjects’ maximal forces (*p* < 0.05) for all grasp types. The comparison of the patients’ sample to subsample H_w in the second MANOVA revealed significantly lower values (*p* < 0.001) in all cases for patients. Slightly lower values appeared for left hands versus right hands, but the difference was significant (*p* < 0.05) only for cylindrical grasp. Table 4 shows the percentage of the decreases in the mean force values per grasp type for HOA patients *versus* healthy women. These differences could be due to the differences between the compared samples’ ages. However when using the normative data presented in^2^, the mean decrease in right hand force for women within the same age range as the HOA patients sample was 30% for cylindrical grasp, 15% for thumb-index pinch and 18% for lateral pinch grasp. Here the decrease obtained for HOA patients was respectively 30%, 55% and 45%, and was similar for cylindrical grasp, but considerably greater for the two pinch grasps.

**Table 4:** Decrease in the % of the mean force values of HOA patients vs. healthy women. Grasp types abbreviations are explained in the caption of Table 1.

Hand	Force decrease (%) of mean force values (HOA patients to healthy women)					
	Cyl (%)	IntPP (%)	LatP (%)	Lum (%)	Obl (%)	TIPinch (%)
Right	42	31	37	37	55	51
Left	42	24	35	30	52	48

**Figure 3:**
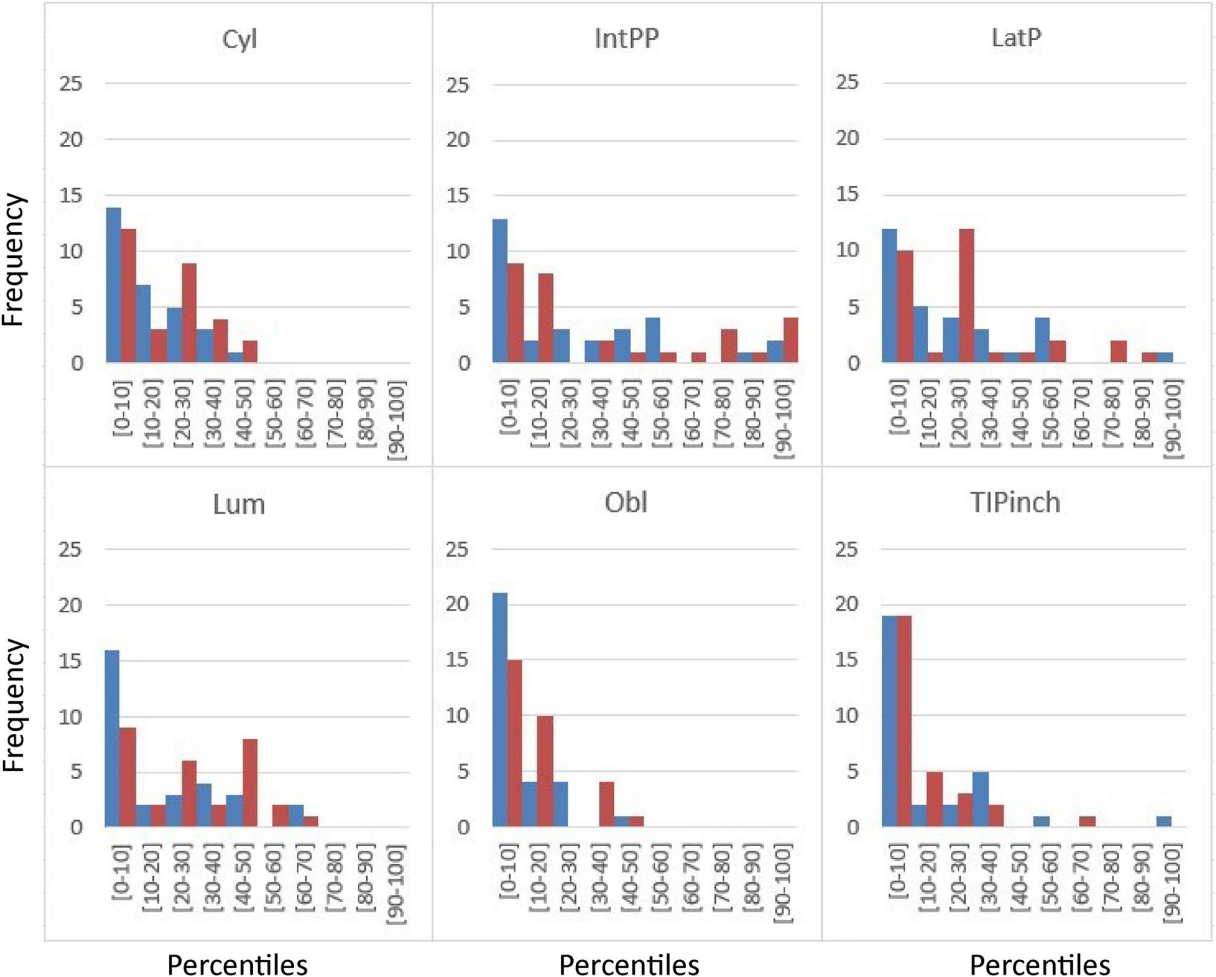
Histogram of the percentiles of force per grasp and hand (blue right hand, red left hand) of the patients belonging to sample H_w. Grasp types abbreviations are explained in the caption of Table 1.

Figure 3 shows the histograms of the percentiles of force values per grasp and hand of the patients as if belonging to sample H_w. Similar results appeared for both hands. Oblique grasp was clearly the one with the lowest percentile values, followed by cylindrical grasp and the two pinch grasps (thumb-index pinch and lateral pinch). The intermediate power-precision seemed to be the least affected. If no effect due to HOA appeared, the histograms would show similar bars for all the percentiles, except for the declining in force expected for the age difference in samples^2^, which would be similar in cylindrical grasp, but the effect is much higher for thumb-index pinch and lateral pinch.

Of the six possible predictor variables used in the step-by-step discriminant analysis (forces for six different grasp types), only one, oblique grasp, was finally entered in the function. For both hands, the Oblique grasp was the only variable included in the resulting discriminant function (Eq. (1) for right hand and Eq. (2) for left hand) when applying discriminant analysis with SPSS-28. This means that a force value lower than 14.413 kg for the right hand or lower than 13.188 kg for the left hand might be inferred as an indicator of having HOA pathology. The right-hand threshold was able to predict the assignment of the subjects participating in the experiment with a success ratio of 85.1% (88.2% for healthy subject, 83.3% for patients), and with the same values after the cross validation. Therefore, the specificity of detection (percentage of participants who truly did not have the disease and were correctly identified) was 88.2%, while sensitivity (percentage of participants who truly had the disease and were correctly identified) was 83.3%. The left-hand threshold was able to predict the assignment of the subjects participating in the experiment with a success ratio of 87.2% (94.1% for healthy subjects, 83.3% for patients), and of 85.1% (88.2% for healthy subjects, i.e., specificity, and 83.3% for patients, i.e., sensitivity) after the cross validation.

1 $$\begin{array}{*{20}c} {HOA\, patient\, if\, Force\, in\, Obl\, \left( {right\, hand} \right) < 14.41\, kg} \\ {Healthy\, otherwise} \\ \end{array}$$

2 $$\begin{array}{*{20}c} {HOA\, patient\, if\, Force\, in\ , Obl\, \left( {left\, hand} \right) < 13.19\, kg} \\ {Healthy\, otherwise} \\ \end{array}$$

## Discussion

This work presents, as a novelty. maximal force values in different grasp types for healthy subjects (segregated by gender) and HOA female patients, namely: lumbrical, intermediate power-precision and oblique grasps, in addition to those commonly provided: cylindrical grasp, thumb-index pad-to-pad and lateral pinch. These values are relevant for functionality^14^. Those of the commonly reported grasps (cylindrical grasp, thumb-index pinch, lateral pinch) are in accordance with the literature^2,7,9^. The thumb-index pinch and lateral pinch forces for HOA patients are similar to those reported by Villafañe et al.^25^, while the cylindrical grasp forces are slightly lower than those reported by Haugen et al.^18^, probably due to the older average age of our sample of HOA women.

The validity of using a Jamar dynamometer to measure lumbrical, oblique and intermediate power precision grasps was checked with correlation coefficients (higher than 0.73) and the intraclass coefficient of the three repetitions performed (higher than 9.92). The correlation coefficients for the commonly measured grasps were similar to those reported in the bibliography for healthy participants^24^, and also to the ICC values of pinch strength in HOA patients^25^, and were of the same order of magnitude for the new grasp types recorded (lumbrical, oblique and intermediate power precision). Furthermore, intrasession repeatability errors were small (7–21%) and of the same order of magnitude for both HOA and healthy participants, and also for the new grasp types recorded than for those commonly reported (cylindrical grasp, thumb-index pinch and lateral pinch). So, the procedure herein described to record maximal grasping forces in these new types of grasps with a Jamar dynamometer is reliable.

Previous works recommend three measurement repetitions for healthy subjects to ensure reliability. The results of the ANOVAs (with repetition as the factor) showed that fatigue did not appear as declining force in subsequent repetitions. Although significant differences (*p* < 0.05) were found in patients’ right hands for oblique and lumbrical grasps, the force in the third repetition was greater. Significant differences (*p* < 0.05) also appeared in healthy subjects’ right hands, which corresponded to a higher value in the second and third repetitions than in the first one for intermediate power precision grasp, and to increasing force with the repetitions for lateral pinch grasp. With no clear decreasing trend with repetitions, these significant differences do not suggest signs of fatigue^26^, nor in healthy individuals, neither in patients. Increasing force in the last repetition could be attributed to an increasing confidence in less intuitive grasps, and despite the images provided, in an attempt to achieve to maximal force in their third attempt, or to any other spurious cause. Werle et al.^7^ found a slight fatigue effect on grip strength. However, those authors only considered a 15-s rest between trials, while our work applied 30-s rests by ensuring 1 min of rest for a given hand because of the alternation of measurements between hands^25^.

The percentiles of force values per grasp and hand of the patients as if belonging to sample H_w (Fig. 3) clearly showed that patients experienced a greater reduction in force capabilities. In fact, significant differences (*p* < 0.05) were found for all the grasp types when comparing healthy and HOA women. A drop in HOA patients’ mean values (Table 4) for the new reported grasps (lumbrical, oblique and intermediate power precision) went from 24% (intermediate power precision left hand) to 55% (oblique grasp right hand), and were of the same order of magnitude as the decrease in commonly reported grasps (cylindrical grasp, thumb-index pinch, lateral pinch), which went from 35% (lateral pinch left hand) to 51% (thumb-index pinch right hand). This decrease was much more marked than that expected only for age reasons^2^ in the grasps in which the thumb is mostly involved. Furthermore, the drop in the force values reported in^2^ for participants aged over 60 years could be due partly to the presence of HOA because of its high prevalence in elderly people, especially women^16^. This is in accordance with HOA patients aged < 60 obtaining lower values than normative ones, but with similar values for those aged > 60^18^.

As HOA can affect different joints, interpreting its impact on hand function is complicated. This is why Lee et al.^20^ analysed the relation between affected joints (corroborated by image) and cylindrical and pinch strength, and with the Disability Arm Shoulder and Hand (DASH) test. They found that the middle finger contributed more to cylindrical strength, and the thumb was an independent predictor. In our work, oblique grasp seems a more significant grasp for HOA detection than analysing cylindrical strength, no matter the affected joints or disease stage. No clear relation with function was found in^20^, probably because DASH is a global test that includes arms and shoulders, while HOA focuses on hands. The relation of HOA with function would benefit from understanding why certain tasks are problematic for HOA patients to better manage this disease^27^ with better rehabilitation processes and recommendations. This paper claims that loss of force in different grasp types can explain problems in performing certain activities usually reported by HOA patients. Thus, detecting a decrease in these forces could not only point out the existence of HOA, but could also serve to infer which tasks may be problematic for patients. Hence, loss in grip and pinch force can result in problems to perform certain tasks that are reported as difficult, which often need force and fine manipulation, as in wringing out washcloths, opening jars or doing up buttons^22^. Yet loss in force for other grasp types, e.g. intermediate power precision or oblique grasp, can explain problems with tasks like using a toothbrush or cutting food with a knife, which are commonly reported^21^. Furthermore, loss of lumbrical grasp force can explain problems with taking out heavy trays out of ovens. In fact, some of the most difficult activities reported by our patients were opening jars (involving oblique grasp), removing heavy trays from ovens (involving lumbrical grasp), and activities that require thumb-index pinch or lateral pinch, like doing up buttons or using sewing needles. All these grasp types involved a major decrease in force compared to healthy women.

The decrease in the forces of different grasp types can be studied to better understand difficulties in performing ADLs, but also to look for a non-invasive detection method. As mentioned in^28^, decreases in elderly people’s grasping force may provide valuable information for clinical diagnosis purposes. This paper proved that just with oblique grasp force, which reduced the most in HOA patients (Fig. 3), may suffice to discriminate with high specificity (88.2%) and sensitivity (83.3%) patients with HOA by using either oblique grasp force of their right hand (OBL(Right) < 14.413) or their left hand (OBL(Left) < 13.188). A clearly negative correlation between HOA disease severity and grip strength has been previously reported^29^, but the equation provided in this work showed that oblique grasp was much more significant to detect HOA than cylindrical grasp, and was able to detect this disease even in different disease stages in the HOA patients sample. The observed results could be also attributed to the affected joints in the osteoarthritis patients’ sample, which was not controlled but was varied. The proposed method could potentially serve as a non-invasive detection tool, indicating the need for subsequent imaging diagnosis, which is not recommended unless there is a need to rule out other diseases^30^. Finally, during the study, a subset of participants underwent sEMG recording while exerting maximum force using various grasps and sEMG recordings were analysed^31^. The sEMG data yielded valuable information and could be potentially used for differentiation purposes. However, it is important to note that the clinical applicability of sEMG falls significantly short when compared to the method proposed in this paper. Future studies should focus on investigating the impact of different types of hand osteoarthritis on grip force and how each type of HOA interacts across various grasp types.

## Conclusion

This study provides grip force data on six grasp types for healthy participants disaggregated by gender and for women with HOA. Notably, data corresponding to three out of the six grasp types constitute a novel contribution that may be of interest in the fields of clinical research, rehabilitation, ergonomics or in design of assistive devices. Comparison of grip forces between healthy and HOA women reveals that women with HOA have reduced strength in all grasp types, to a greater or lesser degree, which may improve the understanding of the functional limitations they suffer. Furthermore, oblique grip strength alone may be sufficient as a further indicator of HOA pathology. This last finding suggests a practical tool for clinicians, which, while not intended for diagnostic purposes, can assist in reinforcing decisions regarding the need of additional tests or imaging diagnostics to ensure appropriate treatment for the patient.

## Data Availability

The datasets used and/or analysed during the current study available from the corresponding author on reasonable request.
